# Obituary

**Published:** 1916-06

**Authors:** 


### OBITUARY

JACOB WESLEY GREEN. Doctor Green, the well known advocate of better impression taking by the use of modeling compound especially, died February 27, 1916. Dr. Green spent practically all his time for the past ten years attending dental meetings and giving clinics on impression methods. He also wrote a small book describing his methods and gave instructions to private classes. His older brother was an expert in prosthetic dental practice and the two worked together in perfecting the methods which have since become very generally accepted. Dr. Green was a very fluent talker, but not consecutive or sequential in his thinking, and consequently did not exert the influence his worth and knowledge of his subject entitled him to do. His enthusiasm was so great that he always attracted attention and he did much to call attention to a feature of practice that has been sadly neglected. He deserves great credit for his loyalty, and the influence of his instruction will make for better prosthetic dentistry in the years to come.

